# Inflammatory Profile and Risk of Post-Intervention Infection in Relation to Myocardial Necrosis Markers

**DOI:** 10.3390/healthcare13182371

**Published:** 2025-09-21

**Authors:** Alexandra Manuela Buzle, Larisa Renata Pantea-Roșan, Mădălina Ioana Moisi, Priscilla Matache, Marc Cristian Ghitea, Evelin Claudia Ghitea, Maria Flavia Gîtea, Timea Claudia Ghitea, Mircea Ioachim Popescu

**Affiliations:** 1 Department of Medical Disciplines, Faculty of Medicine and Pharmacy, University of Oradea, 410073 Oradea, Romania; buzlealexandramanuela@student.uoradea.ro (A.M.B.); larisa.rosan@didactic.uoradea.ro (L.R.P.-R.); procardia_oradea@yahoo.com (M.I.P.); 2 Department of Preclinical Discipline, Faculty of Medicine and Pharmacy, University of Oradea, 410068 Oradea, Romania; moisi.madalina.ioana@didactic.uoradea.ro (M.I.M.); priscilla_pasc@uoradea.ro (P.M.); 3 Faculty of Medicine and Pharmacy, University of Oradea, 410068 Oradea, Romania; ghitea.marccristian@student.uoradea.ro (M.C.G.); ghitea.evelinclaudia@student.uoradea.ro (E.C.G.); gitea.mariaflavia@student.uoradea.ro (M.F.G.); 4 Pharmacy Department, Faculty of Medicine and Pharmacy, University of Oradea, 410068 Oradea, Romania

**Keywords:** acute coronary syndrome, percutaneous coronary intervention, hs-cTn infection, CRP, ESR, NT-proBNP

## Abstract

**Background:** Post-procedural infection worsens outcomes in acute coronary syndrome (ACS). High-sensitivity cardiac troponin (hs-cTn) reflects myocardial injury, but its utility for infection risk prediction after percutaneous coronary intervention (PCI) is uncertain. Objective: This study aimed to evaluate whether high-sensitivity troponin (hs-cTn) levels are associated with the risk of infection and systemic inflammation. **Methods:** We performed an exploratory pilot study of consecutive ACS patients undergoing PCI (*n* = 181) at a tertiary interventional cardiology unit in Romania. Herein, hs-cTn was measured at 24- and 48-h post-PCI. The primary outcome was in-hospital infection (clinical and/or microbiological documentation), with the acknowledgment that nearly half were clinically diagnosed without microbiological confirmation. We assessed discrimination for hs-cTn48h using ROC analysis and explored associations with systemic markers (CRP, ESR, and leukocytes) and NT-proBNP using Spearman correlations. **Results:** Infections occurred in 9/181 patients (5.0%; 95% CI, 2.6–9.2). Notably, hs-cTn48h showed AUC = 0.49 (approx. 95% CI, 0.30–0.68) for infection discrimination. Correlations between hs-cTn48h and inflammatory markers were weak and non-significant (CRP ρ = 0.126, *p* = 0.091; ESR ρ = 0.119, *p* = 0.111; fibrinogen ρ = 0.134, *p* = 0.073), whereas hs-cTn48h correlated modestly with NT-proBNP (ρ = 0.232, *p* = 0.002). **Conclusions:** In this cohort, hs-cTn48h did not predict in-hospital infection after PCI in ACS. These negative findings highlight that troponin should be interpreted primarily as a marker of myocardial necrosis, not infectious risk. Larger multicenter studies with microbiological adjudication and broader biomarker panels are warranted.

## 1. Introduction

Nosocomial and post-interventional infections remain important complications in patients with acute coronary syndrome (ACS) undergoing percutaneous coronary intervention (PCI), contributing to higher morbidity, prolonged hospitalization, and increased costs [1,2]. Surveillance studies report that healthcare-associated infections (HAIs) occur in 3–6% of ACS patients post-PCI, depending on case mix and surveillance criteria [3,4].

According to the Centers for Disease Control and Prevention/National Healthcare Safety Network (CDC/NHSN), HAIs are defined as infections not present on admission and meeting standardized clinical, laboratory, and/or radiologic criteria, including bloodstream infection (BSI), pneumonia, urinary tract infection (UTI), and surgical site infection (SSI) [CDC/NHSN 2023]. Adoption of these definitions reduces diagnostic variability and allows comparability across cohorts.

High-sensitivity cardiac troponin (hs-cTn) is the biomarker of choice for detecting myocardial necrosis in ACS [5,6]. Beyond its role in quantifying ischemic injury, prior studies have suggested that elevated troponin may also reflect systemic inflammation or critical illness. For example, troponin elevations have been described in influenza [7] and COVID-19 [8], where they correlated with systemic inflammatory activation rather than isolated cardiac injury [9,10]. This raises the hypothesis that troponin might integrate myocardial and immune responses and thus signal vulnerability to infection [11,12].

However, in the PCI setting, evidence is conflicting. A multicenter Chinese cohort of non–ST-elevation ACS patients undergoing PCI [13] identified infection as an adverse prognostic event, while another study developed a risk score for infection in STEMI patients post-PCI [14] but did not include troponin as a predictor. These data suggest that infection risk after PCI is real and clinically relevant, yet whether hs-cTn contributes meaningfully to risk stratification remains uncertain [15,16].

Understanding the relationship between hs-cTn and infection risk could refine early monitoring and guide supportive care. At the same time, it is important to distinguish between systemic inflammation, which may accompany myocardial injury, from true infection, which represents a separate pathological process with specific diagnostic criteria [17,18,19,20,21].

Elevated hs-cTn levels have been observed not only in ACS but also in systemic infections such as influenza and COVID-19, where they are associated with adverse outcomes and systemic inflammatory responses [7,8]. This overlap suggests that myocardial injury biomarkers may reflect both ischemic burden and immune activation, potentially linking infarct size and infection risk. However, evidence is conflicting, with some studies reporting strong associations between troponin and systemic inflammation, while others attribute troponin elevation solely to myocardial necrosis. Clarifying this relationship is clinically relevant for early risk stratification and [15,16], infection surveillance in ACS patients post-PCI.

The aim of this study was to evaluate whether hs-cTn levels measured at 24- and 48-h post-PCI are associated with in-hospital infection in ACS patients, using CDC/NHSN-aligned infection definitions. In addition, we assessed correlations with classical inflammatory markers (CRP, ESR, and leukocyte count [17,18,19,20,21]) to explore whether troponin reflects systemic immune activation beyond myocardial necrosis.

## 2. Materials and Methods

### 2.1. Study Design and Setting

This was a retrospective, single-center pilot study conducted at the Interventional Cardiology Unit of the University of Oradea Clinical Emergency Hospital (Oradea, Romania). This study included consecutive patients admitted with acute coronary syndrome (ACS) and treated with percutaneous coronary intervention (PCI) between January 2023 and December 2024.

The study protocol was approved by the Institutional Review Board (approval no. 2379/21 January 2025). Given the retrospective design and use of anonymized data, a waiver of individual informed consent was granted.

### 2.2. Study Population

Inclusion criteria:

Age ≥ 18 years;

ACS diagnosis confirmed by clinical presentation, ECG findings, and elevated hs-cTn above the 99th-percentile upper reference limit (URL), and undergoing successful PCI during the index hospitalization.

Exclusion criteria:

Active infection or fever at admission;
Autoimmune disease, active malignancy, or immunosuppressive therapy;
Missing hs-cTn or inflammatory marker values at 24-h or 48-h post-PCI;
Major complications (e.g., surgery and mechanical circulatory support) are likely to confound the inflammatory status.

**Figure 1:**
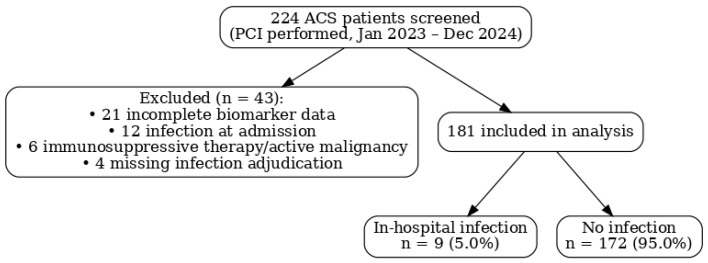
Flow diagram.

STROBE flow diagram: A total of 224 patients with ACS undergoing PCI were screened. Of these, 43 were excluded (21 had incomplete biomarker data, 12 had infection at admission, 6 were on immunosuppressive therapy, and 4 had missing infection adjudication data), leaving 181 patients for the final analysis (Figure 1).

### 2.3. Outcome Definition: In-Hospital Infection

The primary outcome was any in-hospital infection occurring between PCI and discharge (median surveillance window: 7 days, IQR 5–10).

Infections were defined using CDC/NHSN criteria (2023) and classified as follows:

Bloodstream infection (BSI);
Pneumonia;
Urinary tract infection (UTI);
Surgical site infection (SSI).

Each case was further categorized as a hospital-acquired infection (HAI) or present-on-admission (POA) using NHSN Chapter 2 criteria.

Adjudication: Two independent physicians, blinded to troponin values, reviewed cases. Disagreements were resolved by consensus with a third reviewer. Of the 9 infections, 5 (55%) were microbiologically confirmed, while 4 (45%) were diagnosed clinically using CDC/NHSN-compatible criteria. We acknowledge that the reliance on clinical diagnosis without microbiological confirmation in nearly half of the cases increases the risk of outcome misclassification.

### 2.4. Index Test and Laboratory Assays

High-sensitivity cardiac troponin (hs-cTn) was measured using the Roche Elecsys hs-cTnT (Roche Diagnostics GmbH, Mannheim, Germany) assay (5th generation), with the following analytical characteristics:

Limit of detection: 3 ng/L;
99th-percentile URL: 14 ng/L;
Coefficient of variation (CV) <10% at the URL.

Here, hs-cTn was collected at 24 ± 4 h and 48 ± 4 h after PCI completion (sheath removal) and expressed in ng/L.

Other biomarkers:

C-reactive protein (CRP, mg/dL);
Erythrocyte sedimentation rate (ESR, mm/h);
Leukocyte count (×10^3^/µL);
NT-proBNP (pg/mL).

### 2.5. Covariates and Confounding

Potential confounders were pre-specified based on prior PCI infection risk models [18,19]:

Demographics (age and sex);
Clinical characteristics (ACS type, Killip class, diabetes, chronic kidney disease [eGFR], and hemodynamic shock);
Procedural characteristics (vascular access site, number of stents, procedure duration, and contrast volume);
Peri-procedural exposures (mechanical ventilation, central venous or urinary catheters, and prophylactic antibiotics).

Missing data were <5% for all variables and were handled by complete case analysis.

Due to the very low number of infection events (*n* = 9), multivariable adjustment was not feasible (events per variable < 10). Only univariate models were performed, and we emphasize that these exploratory analyses cannot account for confounding. Future studies should apply penalized regression or propensity methods to address this limitation.

### 2.6. Sample Size Considerations

Of the 181 patients, 9 (5.0%) developed infections. Given the events per variable (EPV) < 10, multivariable logistic regression would risk overfitting. We therefore restricted analyses to descriptive comparisons, univariate logistic regression, and ROC evaluation, and this study was explicitly framed as hypothesis-generating. The limited number of events is acknowledged as a major limitation, and future studies with larger cohorts are warranted [22].

### 2.7. Statistical Analysis

All analyses were performed using SPSS v30 (IBM Corp) and Python 3.10 statsmodels.

Descriptive data are presented as mean ± SD or median (IQR) for continuous variables and as *n* (%) for categorical variables.

Group comparisons (infected vs. non-infected) were made using the Mann–Whitney U test for continuous variables and Fisher’s exact test for categorical variables. Effect sizes were reported as mean/median differences with 95% CIs or odds ratios (ORs) with 95% CIs.

Binary logistic regression (univariate) was used to evaluate the association between hs-cTn and infection, limited to univariate models only due to sample size constraints.

ROC analysis was performed for hs-cTn48h, with AUC and bootstrapped 95% CIs (2000 resamples). Calibration was not formally assessed due to limited events and should be evaluated in larger cohorts.

Correlations between hs-cTn and other biomarkers were evaluated using Spearman’s ρ with 95% CI.

A *p*-value < 0.05 was considered statistically significant.

## 3. Results

### 3.1. Study Population and Flow

A total of 224 patients with ACS undergoing PCI were screened between January 2023 and December 2024; 43 were excluded (21 with incomplete biomarker data, 12 with infection at admission, 6 receiving immunosuppressive therapy/with active malignancy, and 4 missing infection adjudication), leaving 181 patients for analysis (Figure 1). In-hospital infections occurred in 9/181 (5.0%); 172/181 (95.0%) had no infection. Given the small number of events, results are presented descriptively and should be interpreted as exploratory.

### 3.2. Demographics and Baseline Characteristics

The study population included 181 patients diagnosed with acute coronary syndrome (ACS) and treated with percutaneous coronary intervention (PCI). Of these, 65.7% were male (*n* = 119) and 34.3% were female (*n* = 62). The majority of patients were from urban areas (52.5%), while 47.5% originated from rural regions. The mean age of the cohort was 63 ± 11 years, with a demographic profile for coronary artery disease.

Laboratory data obtained at statistical admission revealed elevated levels of inflammatory and myocardial injury markers. The mean leukocyte count was 12.31 ± 4.15 × 10^3^/μL, and the mean neutrophil count was 9.10 ± 3.79 × 10^3^/μL, both exceeding normal reference ranges and reflecting systemic inflammatory activation. The mean lymphocyte count was 3.63 ± 5.00 × 10^3^/μL, with greater variability.

Regarding myocardial injury, the initial hs-cTn level was markedly elevated, with a mean of 3223.07 ± 8644.81 ng/L, and with acute coronary syndrome. All baseline characteristics and continuous variables showed statistically significant deviation from the normal reference distribution (*p* < 0.01), as confirmed using *t*-tests.

**Figure 2:**
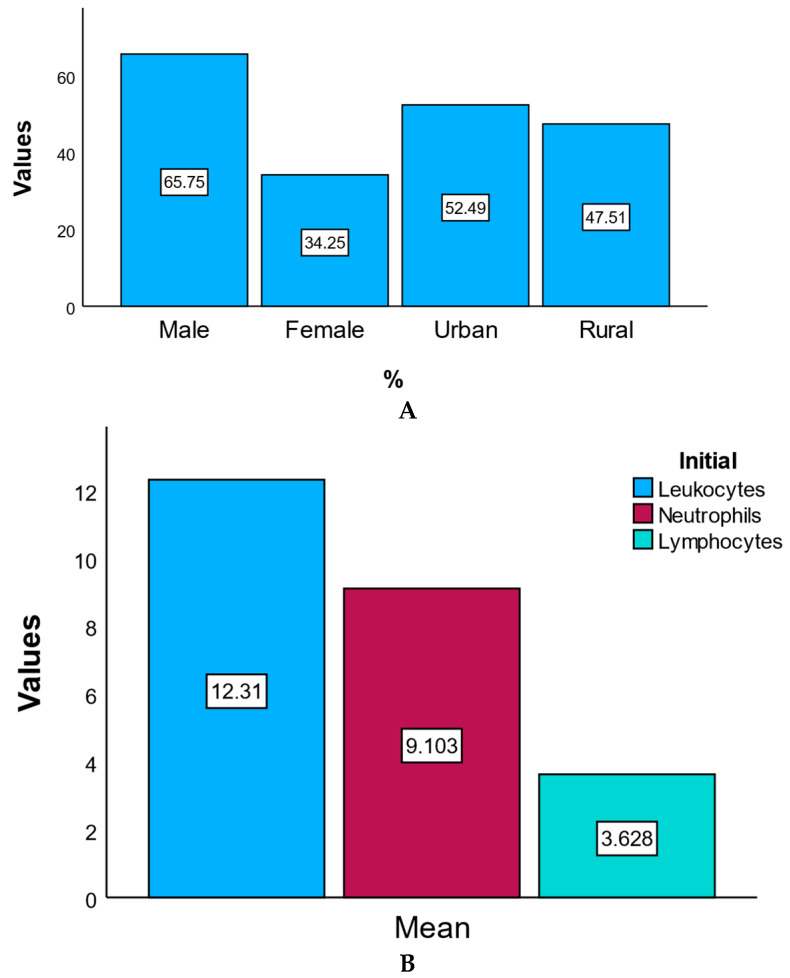
Demographics and baseline characteristics. (**A**) Percentage distribution of patients by gender and area of residence. This bar chart illustrates the demographic distribution of the study population by gender (male and female) and residence (urban and rural), expressed as percentages. (**B**) Mean initial values of leukocytes, neutrophils, and lymphocytes at admission. This chart statistic shows the mean initial values (×10^3^/μL) of key inflammatory markers at hospital admission, including leukocytes, neutrophils, and lymphocytes.

Figure 2 presents the percentage distribution of patients by gender and area of residence (Panel A), as well as the mean initial values of leukocytes, neutrophils, and lymphocytes at hospital admission (Panel B). Panel A illustrates the demographic profile of the study cohort (male vs. female; urban vs. rural), while Panel B summarizes the baseline inflammatory status, expressed as mean cell counts (×10^3^/μL). Essentially, equivalent to the random chance of the population characteristics and initial immune profile.

### 3.3. Discrimination of hs-cTn48h for Infection

**Figure 3:**
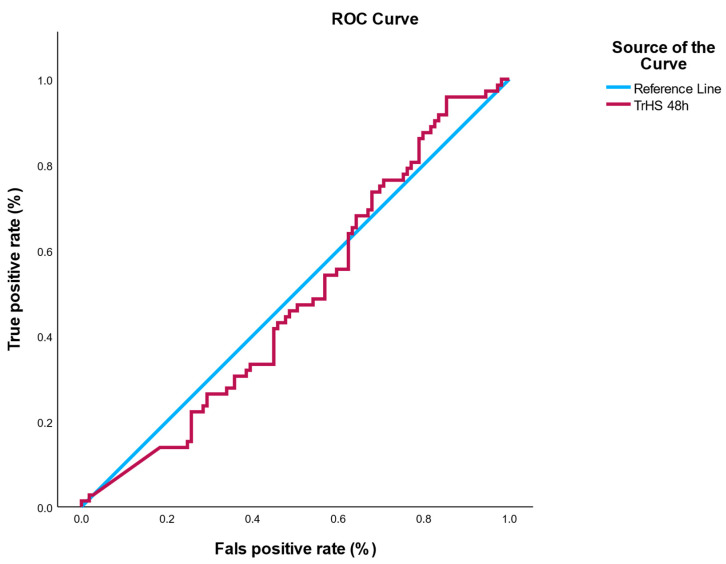
ROC curve for hs-cTn48h predicting in-hospital infection. ROC analysis demonstrated no discriminatory power (AUC = 0.49, 95% CI: 0.30–0.68). The shaded area represents the 95% confidence interval, based on 2000 bootstrap resamples.

Receiver operating characteristic (ROC) analysis for hs-cTn48h yielded AUC = 0.49 (95% CI, 0.30–0.68), indicating no discrimination, essentially equivalent to chance. The Youden-optimal cutoff in this cohort was ~1026 ng/L, but the corresponding sensitivity/specificity values were low and offered no clinical utility (Figure 3). A full sensitivity/specificity table across all observed thresholds is provided in Supplementary Table S1.

### 3.4. Troponin Distribution by Affected Coronary Artery

This subsection analyzes the relationship between troponin levels and the location of coronary artery lesions. The bar chart displays the mean hs-cTn values for patients with involvement of the anterior descending artery, right-statistic coronary artery, and circumflex artery. Among these, the anterior descending artery (ADA) showed the highest mean troponin levels, reflecting the greater extent of myocardial damage typically associated with its occlusion.

This pattern is consistent with the anatomical role of the ADA in supplying a large portion of the anterior left ventricular wall, which, when compromised, results in extensive myocardial necrosis and significantly elevated troponin levels. In contrast, lesions in the right coronary artery (RCA) and the circumflex artery (Cx) were associated with lower mean troponin values, suggesting either smaller infarct areas or more collateral circulation in these territories.

**Figure 4:**
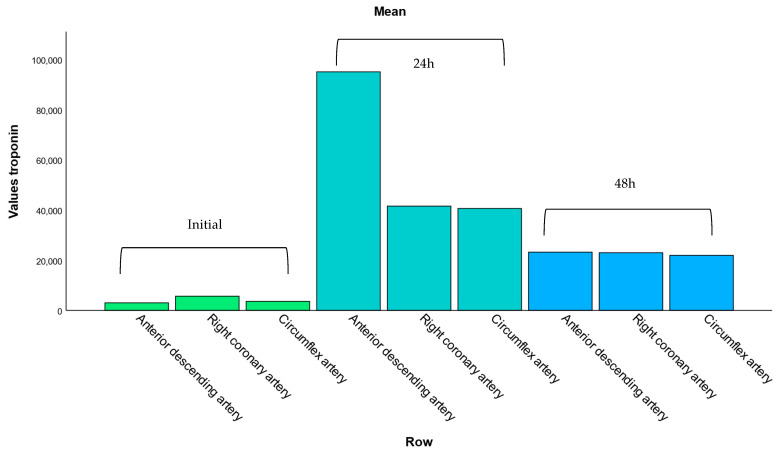
Mean troponin levels according to affected coronary artery. This bar chart displays the average hs-cTn values associated with lesions in the anterior descending, right coronary, and circumflex arteries. Elevated troponin levels are most pronounced in patients with anterior descending artery involvement, consistent with its role in supplying a larger portion of the left ventricle.

Additionally, the repetition of artery names in Figure 4 likely reflects differences in lesion contexts, such as primary versus secondary involvement or single- versus multivessel disease. This reinforces the clinical importance of lesion location in predicting myocardial injury severity and the need for stratified post-intervention care.

### 3.5. ROC Analysis: hs-cTn as a Predictor of Infectious Risk

To evaluate the potential of hs-cTn as a biomarker for post-interventional infection, a receiver operating characteristic (ROC) analysis was conducted using the 48-h post-PCI troponin values (hs-cTn48h). The analysis revealed an area under the ROC curve (AUC) of 0.49, indicating a lack of discriminatory power—essentially equivalent to random chance. Logistic regression models were univariate, evaluating hs-cTn24h, hs-cTn48h, CRP, ESR, and leukocytes individually as predictors of infection.

The optimal cutoff point suggested by the Youden index was 1026.4 ng/L. However, predictive performance at this threshold was clinically irrelevant, with poor sensitivity and specificity values that do not support its use in risk stratification.

The AUC for hs-cTn48h was 0.49, suggesting that troponin levels at 48-h post-PCI, rather than showing no association.

These findings suggest that, in this cohort, elevated troponin levels at 48-h post-PCI are not associated with an increased risk of infection. Despite their role as reliable markers of myocardial necrosis, hs-cTn values appear insufficient for predicting susceptibility to infectious complications in the acute post-procedural phase.

### 3.6. Correlations Between hs-cTn and Systemic Biomarkers

**Figure 5:**
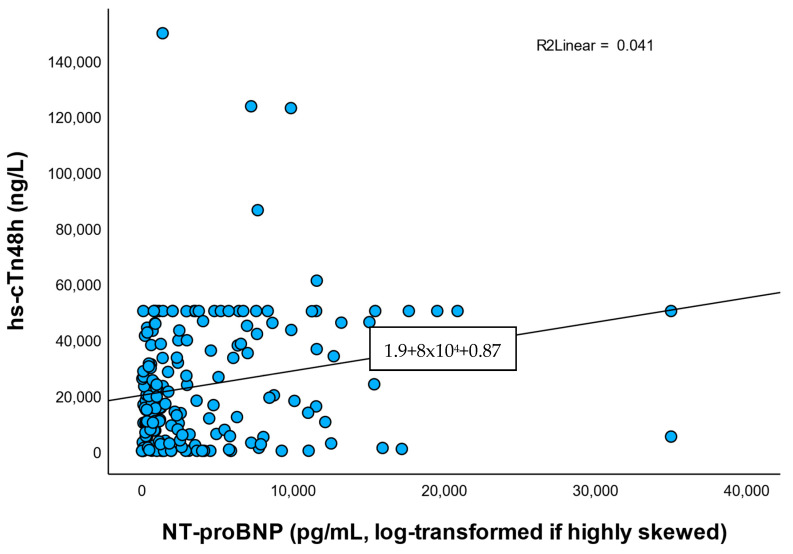
Correlation between hs-cTn48h and NT-proBNP. Scatterplot with LOWESS smoothing demonstrates a modest positive correlation (ρ = 0.23 and *p* = 0.002). Correlation may be confounded through left ventricular dysfunction and renal function.

Notably, hs-cTn48h correlated weakly and non-significantly with CRP (ρ ≈ 0.13), ESR (ρ ≈ 0.12), and fibrinogen (ρ ≈ 0.13), but correlated modestly with NT-proBNP (ρ ≈ 0.23, *p* = 0.002). A scatter plot with LOWESS smoothing is provided in Figure 5. Because NT-proBNP is influenced by left ventricular (LV) dysfunction and renal function, this association may be confounded; we therefore report NT-proBNP alongside eGFR in Table 1 and recommend adjusted analysis in future larger cohorts.

In contrast, weak and nonsignificant-statistic positive correlations were found between hs-cTn48h and inflammatory markers:

- CRP: ρ = 0.126, *p* = 0.091;
- ESR: ρ = 0.119, *p* = 0.111;
- Fibrinogen: ρ = 0.134, *p* = 0.073;
- NT-proBNP: ρ = 0.232, *p* = 0.002.

Correlations with inflammatory markers were weak and non-significant, while hs-cTn correlated modestly with NT-proBNP. This may reflect the link between myocardial injury and ventricular stress rather than systemic infection.

Although none of these correlations reached statistical significance, their direction suggests a potential low-grade systemic inflammatory response accompanying myocardial necrosis.

Taken together, the results indicate that NT-proBNP is more robustly associated with the extent of myocardial damage than inflammatory markers in the immediate post-PCI period. Troponin appears to reflect functional myocardial stress rather than systemic inflammation or risk of infection.

**Figure 6:**
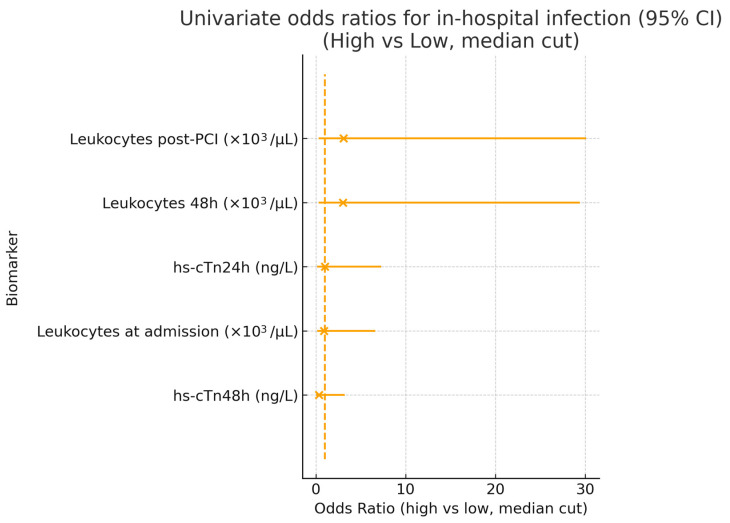
Univariate odds ratios for in-hospital infection. Forest plot displaying odds ratios (OR) with 95% confidence intervals for selected biomarkers, comparing high vs. low values (defined by a median split). The vertical dashed line represents the null value (OR = 1). While elevated leukocyte counts showed numerically higher ORs, all estimates had wide confidence intervals and crossed unity, indicating no statistically significant associations. Both hs-cTn24h and hs-cTn48h demonstrated no predictive value for infection risk in this cohort. Calibration analysis was omitted due to insufficient events.

Univariate odds ratios for selected biomarkers (hs-cTn24h, hs-cTn48h, and leukocyte counts at admission, post-PCI, and 48 h) are shown in Figure 6. Odds ratios had wide confidence intervals crossing unity, consistent with the underpowered sample. Although point estimates for leukocyte counts suggested a higher risk of infection in the “high” group, confidence intervals were extremely wide and crossed unity, reflecting the low number of infection events. Both hs-cTn24h and hs-cTn48h showed no significant association with infection risk.

## 4. Discussion

In this pilot cohort of ACS patients undergoing PCI, hs-cTn levels measured at 24 h and 48 h did not predict in-hospital infection. ROC analysis for hs-cTn48h yielded an AUC of 0.49 (95% CI, 0.30–0.68), indicating non-informative performance and no discriminatory ability [12,23,24,25,26,27]. These findings emphasize that troponin remains a marker of myocardial injury [28,29,30,31] rather than infectious complications in the post-PCI setting [24,25,26,27,28,29,30,31,32,33,34].

Our results align with the established role of hs-cTn as the gold standard biomarker of myocardial necrosis, quantifying the extent of ischemia and infarction [5,6]. In contrast, in critically ill or septic populations, elevated troponin is frequently observed and reflects systemic illness [34], inflammatory cytokine storm, and microvascular dysfunction [14], rather than primary infection risk [35]. This distinction is important: while troponin elevation may serve as a poor prognostic marker in sepsis or severe infections, in ACS patients it reflects cardiomyocyte necrosis rather than immune activation, and thus fails to serve as an infection predictor.

Our results are consistent with the established literature:

In sepsis and critical illness, troponin elevations are common and correlate with severity or mortality before adjustment, but often lose independent predictive value after controlling for confounders [28,36,37].

In ACS, validated infection risk scores rely on clinical variables (age, Killip class, albumin, WBC count, vascular access, and device exposure) rather than hs-cTn [21,24]. These models achieve strong discrimination (C-statistic ≈ 0.85), far superior to hs-cTn alone (AUC 0.49 in our study). Our negative findings thus confirm that hs-cTn does not add incremental predictive value to established infection risk models.

Several factors may explain this unexpected finding. First, patients with larger infarcts and higher hs-cTn levels may have received more intensive monitoring, early antibiotic prophylaxis, and invasive care pathways, reducing their infection risk. Second, some infections were likely catheter-associated or community-acquired and not directly related to infarct size or myocardial injury. Third, infection susceptibility may be more strongly driven by frailty, comorbidities (e.g., diabetes and chronic kidney disease), or procedural complexity, rather than the extent of necrosis reflected by troponin levels [23,24,25,26,27].

Previous studies have reported associations between elevated troponin levels and systemic inflammation or infection, particularly in septic or critically ill populations [28,29,30,31]. However, in ACS patients, hs-cTn remains primarily a marker of ischemia and necrosis rather than immune activation [32,33,34].

In this context, troponin alone appears insufficient to stratify infectious risk, reinforcing the multifactorial nature of post-PCI complications.

Mechanistically, this discrepancy may be explained using the different biological origins of troponin release. In ACS, hs-cTn is released due to myocyte necrosis from ischemia-reperfusion injury, independent of infection risk. In systemic infections and sepsis, however, troponin elevations are thought to reflect multifactorial injury mechanisms, including inflammatory cytokine-mediated myocyte apoptosis, endothelial dysfunction, and microcirculatory failure. Therefore, the lack of association between hs-cTn and infection in our study is consistent with the pathophysiological distinction between ischemic necrosis and systemic inflammation [24,25].

Multivariate adjustment for confounders (e.g., age, diabetes, baseline CRP, and renal function) was not performed due to the small number of infection cases, limiting model stability. Future studies with larger sample sizes are required to build adjusted risk models.

Nearly half of infection cases were diagnosed clinically without microbiological confirmation, which may have resulted in diagnostic variability and underestimation or overestimation of infection incidence.

Correlation analyses between hs-cTn and inflammatory markers (CRP, ESR, and leukocyte count) were weak or non-significant, further supporting the notion that myocardial injury and systemic infection are only partially overlapping processes in the early post-procedural phase [14,35]. Infection-specific biomarkers, such as procalcitonin or IL-6, were not measured, limiting mechanistic interpretation. Taken together, these limitations mean our findings should be regarded as hypothesis-generating only. Larger, prospective, multicenter studies with standardized infection definitions and richer biomarker panels are needed to validate these observations.

### 4.1. Possible Explanations

Several factors may explain the absence of predictive value: outcome heterogeneity meaning nearly half of infections were clinically diagnosed without microbiological confirmation, increasing the risk of misclassification; small event count which refer to only nine infections (5%) precluded robust modeling and increased uncertainty in effect estimates; multifactorial risk representing that infection after PCI is more strongly driven by comorbidities, frailty, procedural exposures, and immune status than by infarct size or myocardial injury; clinical practice factors that explain that patients with higher hs-cTn (larger infarcts) may have received more intensive monitoring or early antibiotics, potentially mitigating infection risk.

### 4.2. Study Limitations

This study has several important limitations. Its limited generalizability design restricts causal inference and generalizability. The small sample size (*n* = 181) and low infection incidence (~5%, nine cases) reduced statistical power, limiting the ability to detect subtle associations or build reliable predictive models. Although infections were adjudicated using CDC/NHSN criteria, nearly half were diagnosed clinically without microbiological confirmation, raising the possibility of diagnostic misclassification. Treatment-by-indication bias may also have occurred, as antibiotics could have been initiated for non-infectious fever or prophylaxis. Biomarker assessment was limited to a single hs-cTn assay (Roche Elecsys hs-cTnT, 5th generation), and missing biomarker data (<5%) were handled by complete case analysis, which may introduce bias. In addition, key immunological markers (e.g., procalcitonin, IL-6, and TNF-α) were not measured, limiting mechanistic insight. The absence of such infection-oriented biomarkers in our panel limits mechanistic insight and prevents distinction between sterile inflammation and true infection. Finally, the absence of an external validation cohort prevents assessment of reproducibility. Future multicenter, prospective studies with standardized infection definitions, larger cohorts, and comprehensive biomarker panels are required to confirm and extend these findings.

### 4.3. Clinical Implications

These findings indicate that hs-cTn should not be used as an infection risk marker in ACS patients following PCI. Instead, hs-cTn should continue to be interpreted strictly as a marker of myocardial necrosis, while infection risk assessment should rely on inflammatory biomarkers (e.g., CRP and leukocyte count) and standardized surveillance criteria, integrated with clinical and procedural risk factors. This study should be interpreted as hypothesis-generating rather than definitive.

## 5. Conclusions

In this retrospective pilot cohort of ACS patients undergoing PCI, hs-cTn measured at 24 h and 48 h showed no predictive value for in-hospital infection. ROC analysis confirmed a non-informative AUC (≈0.49), and correlations with inflammatory markers were weak. These findings indicate that hs-cTn should continue to be interpreted solely as a marker of myocardial necrosis rather than infectious risk. Given the small sample size, limited events, and diagnostic variability, these results must be interpreted cautiously and viewed as hypothesis-generating. Larger, prospective studies are necessary before excluding the potential role for hs-cTn in infection risk stratification.

This study demonstrates that hs-cTn48h is not a reliable predictor of infection risk in ACS patients undergoing PCI. These findings challenge the conventional assumption that higher myocardial injury equates to greater infectious vulnerability and emphasize the complexity of infection risk stratification in post-PCI care.

Future multicenter, prospective studies incorporating microbiological adjudication, adjustment for clinical confounders, and expanded biomarker panels (e.g., procalcitonin and IL-6) are required to clarify mechanisms and establish reliable infection risk models after PCI. While hs-cTn remains essential for evaluating ACS severity, its role as an infection biomarker appears limited and should be interpreted with caution.

## Figures and Tables

**Table 1:** Baseline demographic and clinical characteristics stratified using the in-hospital infection status. We report standardized differences rather than *p*-values to reflect imbalancs, given the low number of events.

Parameter	Infection (*n* = 9)	No Infection (*n* = 172)	Std. Diff
Gender, male	6 (66.7%)	113 (65.7%)	0.02
Environment, urban	5 (55.6%)		0.07
Age, years (mean ± SD)	64 ± 12	63 ± 11	0.09
Leukocytes at admission (×10^3^/µL, mean ± SD)	13.2 ± 4.8	12.3 ± 4.1	0.22
Neutrophils at admission (×10^3^/µL, mean ± SD)	9.7 ± 4.0	9.1 ± 3.8	0.16
Lymphocytes at admission (×10^3^/µL, mean ± SD)	3.4 ± 4.8	3.6 ± 5.0	0.04
hs-cTn at admission (ng/L, median [IQR])	2850 [1300–7500]	3200 [1150–8600]	0.11

Std. Diff = standardized difference. hs-cTn = high-sensitivity cardiac troponin.

## Data Availability

All the data processed in this article are part of the research for a doctoral thesis, and are archived in the database of the corresponding author, where the interventions were performed.

## Supplementary Materials

The following supporting information can be downloaded at https://www.mdpi.com/article/10.3390/healthcare13182371/s1. Table S1: Threshold performance of hs-cTn48h for predicting in-hospital infection after PCI.
