# Supplementary material for: Inflammatory Profile and Risk of Post-Intervention Infection in Relation to Myocardial Necrosis Markers
Source: Healthcare (Basel). 2025 Sep 21;13(18):2371. doi: 10.3390/healthcare13182371 (PMC12469657; doi:10.3390/healthcare13182371)
Supplement: Supplementary file 1 [file healthcare-13-02371-s001.zip › healthcare-3829675-supplementary.pdf]

Supplementary Table S1. Threshold performance of hs-cTn48h for predicting in-hospital infection after PCI.

Threshold (ng/L)	TP	FP	TN	FN	Sensitivity	Specificity	PPV	NPV	Youden_J
0	4	172	0	0	1	0	0.022727		0
1	4	131	41	0	1	0.238372	0.02963	1	0.238372
2	3	70	102	1	0.75	0.593023	0.041096	0.990291	0.343023
3	3	16	156	1	0.75	0.906977	0.157895	0.993631	0.656977
4	0	9	163	4	0	0.947674	0	0.976048	-0.05233

TP = true positives; FP = false positives; TN = true negatives; FN = false negatives; PPV = positive predictive value; NPV = negative predictive value; Youden's J = Youden's index (sensitivity + specificity − 1); hs-cTn = high-sensitivity cardiac troponin; PCI = percutaneous coronary intervention.
